# Non-Precious Electrodes for Practical Alkaline Water Electrolysis

**DOI:** 10.3390/ma12081336

**Published:** 2019-04-24

**Authors:** Alejandro N. Colli, Hubert H. Girault, Alberto Battistel

**Affiliations:** 1 Laboratoire d’Electrochimie Physique et Analytique, École Polytechnique Fédérale de Lausanne, EPFL, Valais Wallis, Rue de l’Industrie 17 Case Postale 440, CH-1951 Sion, Switzerland; hubert.girault@epfl.ch (H.H.G.); alberto.battistel@rub.de (A.B.); 2 Universidad Nacional del Litoral, CONICET, Programa de Electroquímica Aplicada e Ingeniería Electroquímica (PRELINE), Facultad de Ingeniería Química, Santiago del Estero 2829, S3000AOM Santa Fe, Argentina

**Keywords:** Alkaline water electrolysis, Raney-Ni, stainless steel 316, equilibrium potential, water splitting, *iR* correction

## Abstract

Water electrolysis is a promising approach to hydrogen production from renewable energy sources. Alkaline water electrolyzers allow using non-noble and low-cost materials. An analysis of common assumptions and experimental conditions (low concentrations, low temperature, low current densities, and short-term experiments) found in the literature is reported. The steps to estimate the reaction overpotentials for hydrogen and oxygen reactions are reported and discussed. The results of some of the most investigated electrocatalysts, namely from the iron group elements (iron, nickel, and cobalt) and chromium are reported. Past findings and recent progress in the development of efficient anode and cathode materials appropriate for large-scale water electrolysis are presented. The experimental work is done involving the direct-current electrolysis of highly concentrated potassium hydroxide solutions at temperatures between 30 and 100 °C, which are closer to industrial applications than what is usually found in literature. Stable cell components and a good performance was achieved using Raney nickel as a cathode and stainless steel 316L as an anode by means of a monopolar cell at 75 °C, which ran for one month at 300 mA cm^−2^. Finally, the proposed catalysts showed a total kinetic overpotential of about 550 mV at 75 °C and 1 A cm^−2^.

## 1. Introduction

Although water electrolysis has been known for around 200 years [1], this technique received special prominence in recent years because of its potential role in the hydrogen energy economy. However, it still contributes only a minor fraction of the total hydrogen production [2]. 

There are two ways to perform water electrolysis, namely, in basic or acidic medium, the latter is made possible by a polymeric membrane (in general Nafion), which acts also as solid electrolyte. Electrolyzers based on this technology offer major advantages over their alkaline counterparts such as greater safety through the absence of a caustic electrolyte, a more compact design due to higher current densities, and higher operating differential pressures. The limited lifetime and the high cost of the membranes and cell components, usually titanium plating to resist corrosion under acidic medium, are important limitations for this technology, as also is the use of expensive precious metal electrocatalysts. Recently it has been shown with a simple physical model, parameterized with experimental data and based on the gas-permeation voltage–current characteristic, and heat balances that alkaline cells could achieve better efficiency than cells with Nafion membranes [3].

Operation in alkaline conditions also unlocks the possibility of using non-precious metal electrocatalysts [4], cheaper cell components, and lower energy consumption, leading to the possibility of cheaper hydrogen production. Existing alkaline water electrolysis plants are based on cells with an aqueous KOH or NaOH (20–40 wt.%) electrolyte and a porous separator at temperatures between 70 and 110 °C and at atmospheric or elevated pressure (up to 30 bar) [1]. The cell and stack costs represent nearly 20%–30% of the total cost of an alkaline electrolyzer. This means that an increase in the cell performances is well sought as long it is balanced by a small cost increase, which favors non-precious metal materials in commercial water electrolysis cells. 

Electrode materials should have good corrosion resistance, high conductivity, and high catalytic activity for the hydrogen evolution reaction (HER) and the oxygen evolution reaction (OER). Besides the catalyst composition, the morphology has a strong influence on both HER and OER, being less important for OER. Polarization curves are the key performance indicators used to evaluate and compare the performances of catalysts for HER and OER; however, polarization curves can differ significantly from one author to another. The literature can be a poor guide to the performance of cathodes and/or anodes for industrial water electrolyzers. Comparisons based on published performance is difficult to do because: (i) much of the literature relates to room temperature and pressure; (ii) the equilibrium potential is not corrected for temperature and strong alkali concentrations, leading to disparity between different authors that use different temperatures and concentrations of supporting electrolytes; (iii) the geometrical area of electrodes when electrode meshes are used; (iv) unclear effect of bubble coverage or *iR* compensation [5]; (v) most of the experiments in the literature are performed at low current densities [6]; and finally and most importantly (vi) the discussion of HER and OER in terms of Tafel slopes and exchange current densities measured in current density ranges far below those employed for industrial water electrolysis. Moreover, it is important to test electrode materials under realistic process conditions for an extended period of time [7].

Another research branch aims at optimizing cell designs to provide high surface areas for catalysts, low cell resistance, proper flow design for the evacuation of gasses, and appropriate current and potential distribution [8,9]. Configuration of water electrolysis cells can go from conventional monopolar tank cells (simple, reliable, and flexible) through bipolar filter press cells (lower ohmic losses and more compact) and finally to the zero-gap design.

In the first part of the present contribution we intend to give guideline for people working in the field about common assumptions usually made that can produce errors in results and confuse readers. Additionally, we report our investigations and various comparisons on some of the most promising electrocatalysts for HER and OER for alkaline electrolyzers found in literature, with the focus on their applications in real industrial devices.

## 2. Materials and Methods 

### 2.1. Common Assumptions

#### 2.1.1. Equilibrium Potential

Although one might think that the calculation of the values of the relevant thermodynamics parameters for various operating condition would be a relatively straightforward matter, serious errors have crept into the literature and have led to a considerable amount of confusion. In contrast to most activity coefficients of uncharged species, salt activity coefficients can significantly differ from unity. As a result, they must be always carefully considered to make proper calculations whenever the chemical potential of an electrolyte in solution is involved.

In the following development it was assumed that there were two distinct compartments, one for hydrogen and one for oxygen evolution, at atmospheric or the same pressure, *p*, as it was usually done in an alkaline electrolyzer through the use of matched pressure relief valves. In each of these compartments, the gaseous solutions were in equilibrium with water in the electrolyte. Also, hydrogen, oxygen, and water vapor were considered as ideal gases, and the first two formed ideal binary gaseous solutions with the third. The partial pressure of water vapor in each of the wet gases will be equal to *p*_w_, the aqueous vapor pressure of the KOH/NaOH solution:

(1) $$p=p_{w}+p_{H_{2}}=p_{w}+p_{O_{2}},$$

Starting from the equations of alkaline water electrolysis:

(2) $$\begin{array}{cc} \mathrm{Anode} & 2\;{\mathrm{OH}}^{-}⇌1/2O_{2}+H_{2}O+2e^{-}, \end{array}$$

(3) $$\begin{array}{cc} \mathrm{Cathode} & 2\;H_{2}O+2e^{-}⇌H_{2}+2\;{\mathrm{OH}}^{-}, \end{array}$$

(4) $$\begin{array}{cc} \mathrm{Total} & H_{2}O⇌H_{2}+1/2\;O_{2}, \end{array}$$

and considering the definition of equilibrium potential for each half reaction, then:

(5) $$\begin{array}{cc} \text{From Equation (2)} & E^{+}=E_{H_{2}{O/O}_{2}}^{0}+\frac{RT}{2F}\ln\left(\frac{a_{H_{2}O}f_{O_{2}}^{1/2}}{a_{{\mathrm{OH}}^{-}}^{2}}\right), \end{array}$$

(6) $$\begin{array}{cc} \text{From Equation (3)} & E^{-}=E_{H_{2}{O/H}_{2}}^{0}-\frac{RT}{2F}\ln\left(\frac{a_{{\mathrm{OH}}^{-}}^{2}f_{H_{2}}}{a_{H_{2}O}^{2}}\right), \end{array}$$

(7) $$\begin{array}{cc} \text{From Equation (4)} & E^{+}-E^{-}=E_{H_{2}{O/O}_{2}}^{0}-E_{H_{2}{O/H}_{2}}^{0}+\frac{RT}{2F}\ln\left(\frac{f_{H_{2}}\;f_{O_{2}}^{1/2}}{a_{H_{2}O}}\right), \end{array}$$

where *E*_0_ is the standard half-cell potential and can be obtained from thermodynamic tables, R is the universal gas constant, F the Faraday constant, *T* the temperature, *a*_i_ the activity of the *i*th specie, and *f*_i_ the fugacity of the *i*th gaseous specie. The activity, *a*_i_, of a given specie can be expressed by: *a*_i_ = *m*_i_*γ*_i_, where *m* is molality (or molarity) and *γ* the activity coefficient.

In order to predict the activity coefficients of strong electrolytes in pure solutions we used the methodology proposed by Kusik and Meissner [10], as it was already shown to be accurate [8,11]. As we considered an ideal gas (experiments are usually done at atmospheric pressure), fugacity can be replaced by the partial pressure of the gases.

**Figure 1:**
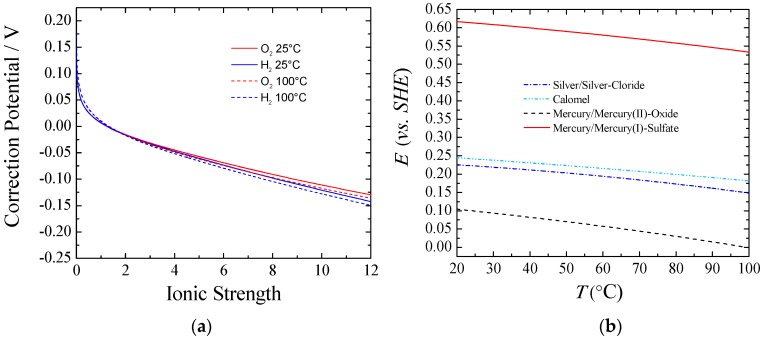
(**a**) Correction of the equilibrium potential from standard conditions vs. ionic strength of KOH. *T* = 25 and 100 °C; *P* = 1 atm.; (**b**) Potential vs. SHE for different reference electrodes against temperature.

Figure 1 part (**a**) shows the equilibrium potential correction at a given temperature that should be applied to the standard conditions when experiments are done under different ionic forces. It can be observed that for ionic strength higher than one, the correction potential from the standard conditions was larger for Equation (6) than for Equation (5), and the difference increased according to the ionic strength. This behavior can be explained taking into account the activity coefficients and water activity. The higher the concentration of KOH/NaOH, the lower the water activity was, and the more Equation (6) and Equation (5) departed from the standard. Also, a higher equilibrium potential for the full reaction, Equation (4), could be observed from Equation (7). Thus, with an increasing the KOH/NaOH concentration, water activity was decreased, viz., a larger voltage was required. However, in practical applications this is a small price to pay to keep the electrolyte resistance as low as possible. In real-world usage, with an electrolyte concentration of about 30% wt. (~7 M), the equilibrium potentials for both the HER and the OER would shift negatively from about 70–90 mV at room temperature. Nevertheless, the voltage to split water would be increased by only 10 mV.

Some authors have shown [12] that hydrogen overpotential is higher than oxygen overpotential, which is somewhat difficult to accept given the fast kinetics for hydrogen evolution and the slow kinetics for oxygen [13]. One of the possible explanations can be the shifting in equilibrium potential due to actual activity coefficients, as explained above.

Figure 1 part (**b**) shows the potential correction vs. the standard hydrogen electrode (SHE) for different common reference electrodes as a function of temperature. Additionally, care must be taken with regards to liquid junction potentials (LJPs), which depends on the reference electrode used for a given electrolyte. Changes in LJPs can be fairly large, especially under the dilution conditions used to determine relative ion permeabilities. Corrections up to 55 mV can be necessary [14]. 

In the present contribution, the actual potentials were corrected for temperature [15], LJP [16], equilibrium potential [10], and reference electrode potential as well (from Figure 1 part (**b**)).

#### 2.1.2. *iR* Correction

In order to investigate the kinetics of an electrochemical reaction, any other source of uncertainty should be reduced. In particular, the ohmic drop, which is the voltage drop due to the resistance of the solution or due to the electrical contacts, is one of the major sources of disturbance. It distorts the cyclic voltammetry and flattens the polarization curves.

One common misconception is that by knowing the ohmic drop, it is possible to correct cyclic voltammetry simply by adding to the potential scale a value taken by multiplying the current and the ohmic drop. This is, in general, a good way for steady-state, or quasi-steady-state techniques such as staircase voltammetries or when the current is directly controlled, e.g., galvanodynamic experiments. In a dynamic experiment like cyclic voltammetry, the effect of the ohmic drop is more subtle: it alters the real scan rate perceived by the electrode interface [17] and a more mathematical involved solution is necessary. Alternatively, if the instrument at hand allows it, the use of an uncompensated resistance correction directly during the experiment is advisable. 

**Figure 2:**
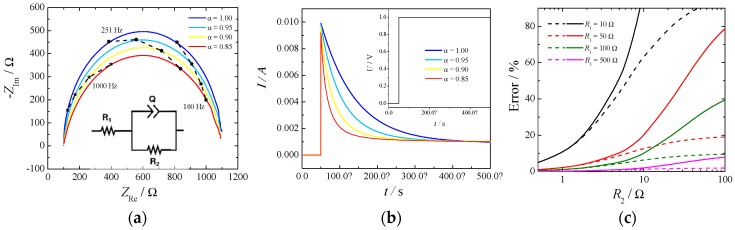
(**a**) Nyquist plot of a typical Randles circuit made with *R*_1_ = 100 Ω, *R*_2_ = 1000 Ω, and a constant phase element (CPE) with Q = 10^−6^ S s^α^. (**b**) Profiles of the current given by the potential step and inset: 1V potential step used to simulate a current-interrupted experiment. (**c**) Error (%) of estimating the cell resistance (*R*_1_) by the current-interrupt method. Q = 10^−6^ S s^α^, α = 1. Measurement done every 10 μs (dashed lines) or 50 μs (full lines).

There are two possibilities to measure the ohmic drop: electrochemical impedance spectroscopy (EIS) and current-interrupt experiments. The first relies on the measurement of the impedance of the electrochemical cell at different frequencies and estimating the ohmic drop as the high frequency intercept of the impedance in the complex plane with the real axis. For a system to be well-conditioned, the impedance should tend toward a real value at high frequency, and it is usually a straightforward method to determine this value. On the other hand, in a current-interrupt measurement the current in the cell is switched off briefly, and the voltage is recorded a few microseconds after the interruption. Both methodologies have their drawbacks, primary EIS require additional circuitry, which is not always included in commercially available potentiostats, while current-interrupt heavily relies on an elevated time resolution required to be able to resolve the current at the exact time of the switching. An example of a simple Randles circuit with a constant phase element (CPE) in place of a standard capacitor is depicted in Figure 2. Part (**a**) shows the Nyquist plot, part (**b**) shows the current decay for a profile of potential when the cell is switched on (Inset), and part (**c**) depicts the errors of estimating the cell resistance, *R*_1_, as a function of the kinetic resistance, *R*_2_, by the current-interrupt method. 

The high frequency intercept was clearly visible in the Nyquist plot (Figure 2 part (**a**)) independently of the constant phase element. However, the slope of the current decay in part (**b**) was influenced by the *α* value of the CPE: the lower the value the higher the slope. In this particular case an uncertainty of 10 μs in reading the exact time of the switching would produce an error in the estimate of the ohmic drop of 10% in the case of α = 1 and of 50% in the case of α = 0.8. The parameter α is usually related to the surface roughness of the electrode [18] and current distribution in the cell [19]. 

Additionally, from Figure 2 part (**c**) it can be seen how the error in estimating the cell resistance, by the current-interrupt method, increases while the measuring time delay and the kinetic resistance increases. This last phenomenon is more important for OER because of the slow reaction rate. Therefore, extrapolation of ohmic drop from current-interrupting should be made with extreme care. 

In our experiments the ohmic drop extracted by EIS and current-interrupted was within a 5%–15% difference when measured in correspondence with HER, but it gave much larger differences when measured in correspondence with OER (nearly 50%–70%).

## 3. Experiment

### 3.1. Electrode Materials and Synthesis

Nickel (Ni) and stainless steel 316L (SS316L) disk electrodes of 3 mm diameter were built from commercially available nickel 99.78% from ADVENT research materials and stainless steel 316L from Shanghai Bozhong Metal Group, respectively. The electrodes were polished with sandpaper (#300–#1500) without significant differences in the obtained results for the different roughness. Aged Ni was obtained by putting the Ni electrode surface in contact with 6–7 M KOH/NaOH and working few hours as anode (OER) or cathode (HER) at a fixed current density (usually >200 mA cm^−2^).

In the case of Raney nickel on Ni-Zn base (Ra-Ni), we have performed electroplating from a modified Watts bath (360 g/L of nickel(II) sulfate hexa/hepta-hydrate for plating, 30 g/L of zinc sulfate heptahydrate 99.5% from Aldrich, and 40 g/L of boric acid 99% from AB) onto Cu substrate. The materials were galvanostatically deposited at a current density of –50 mA cm^−2^ at 55 °C and facing up without stirring during 90 min. After deposition the electrodes were treated for some hours in 6 M KOH solution at room temperature. During this time the electrodes produced hydrogen making the solution sparkling. Several materials were synthetized and tested for HER and OER. Apart Raney nickel (Ra-Ni), also nickel, copper, and cobalt alloys (NiCuCo) were electroplated from a modified Watts bath with 360 g/L of nickel (II) sulfate hexa/hepta-hydrate for plating ( >20.6% Ni and Co basis from Aldrich), 30 g/L of zinc sulfate heptahydrate (99.5% from Aldrich), 20 g/L of Copper(II) sulfate pentahydrate (>98% from Aldrich), and 40 g/L of boric acid (>99.5% from Aldrich) onto a Cu substrate. 

Additionally, porous nickel [20,21], Co, NiFe [22], and Ni-Fe(OH) [23,24] were synthesized following the given references. Also, addition of Co in solution through dissolution of some traces of CoSO_4_ (Co in situ) was tried.

### 3.2. Electrolysis Cell Assembly

**Figure 3:**
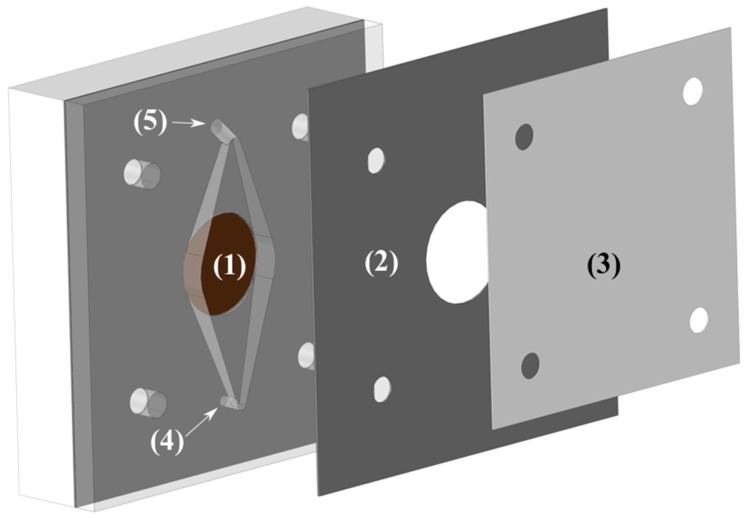
Schematic representation of one half of the cell. (1) Electrode housing. (2) Structural divider. (3) Membrane. (4) Electrolyte inlet port. (5) Electrolyte outlet port.

The cell was made of two pieces of polypropylene (one is shown in Figure 3) with a Zirfon Perl 500 UTP (AGFA) membrane in between. The electrode area was 3.14 cm^2^ and had a 10 mm inter-electrode gap to allow easy flow of electrolyte and gasses. The reactor was part of a flow circuit system consisting of a peristaltic multichannel Ismatec^®^ pump, a reservoir, and connections to maintain the temperature at the preset value, 75 °C. The electrolyte was pumped from a single reservoir of 500 mL through two channels to the cell, and the outlet of both channels was returned to the same reservoir, where the solution was purged with nitrogen. The flow rate was increased until no improvement was read in the cell voltage (***U***_cell_), being the final operational flow rate around 250 mL min^−1^.

### 3.3. Electrochemical Measurements

The electrochemical experiments were conducted with a BioLogic^®^ SP-150 potentiostat (Bio-Logic Science Instruments SAS, Seyssinet-Pariset, France) for the EIS, current-interrupted experiments, and the polarizations with a two-channel power supply for the long-term experiments. In all the experiments, the reference electrode was an Ag|AgCl| 3 M KCl with a double-junction filled with the same electrolyte of the main solution (6 M KOH) to avoid Cl^−^ contaminations. For the polarization experiments, N_2_ bubbling and vigorous stirring were used to avoid bubble coverage and to ensure that the reaction rate was not limited by the mass-transfer (agitation was increased until no change in potential was found). The ohmic resistance was measured for each experiment by impedance spectroscopy, estimated by the intercept of the high frequency arc with the real axis, and *iR* correction was employed to eliminate its effect from the results. Finally, the equilibrium potentials in the given conditions were calculated by using the activity coefficients given by Kusik and Meissner [10], and the respective overpotentials were derived as described in Section 2.1. All experiments were performed at temperatures between 30 and 100 °C in 6 M KOH with a platinum counter electrode.

Two kinds of experiments were performed. The first was a current-controlled polarization where the current was held constant. Once the sample reached steady-state (between 5 and 15 min), the value of the potential vs. the reference was taken. Subsequent current steps were done for the same sample between 5 and 1000 mA cm^−2^. The second kind of experiment was a long-term polarization at 300 mA cm^−2^ at a constant temperature in the electrolysis cell.

## 4. Results and Discussion

### 4.1. Kinetic Behavior

Comparison of the catalyst based on the published performance was difficult, as detailed data on measuring techniques are not given, or various techniques were applied. Operation conditions, such as the temperature, often differed vastly, and different types and concentrations of electrolytes were used.

One of the problems observed with Ni-based materials for water electrolysis was the loss of activity, as indicated by the time variation of the cathode and anode potentials [13]. Thus, instead of a classical quasi-steady-state linear sweep voltammetry, the working electrode was held at a given current density until steady-state was reached. The potentials of the working and counter electrode were monitored against the Ag|AgCl| 3 M KCl reference electrode. The same experiments were then performed at different temperatures for different materials. All the overpotentials were corrected for the shift of potential of the reference electrode and of the equilibrium potential because there were changes in temperature and electrolyte concentrations as well as an *iR* drop.

#### 4.1.1. Hydrogen Evolution Reaction (HER)

Ni-based alloys have been the object of extensive research efforts. Considering pure metals, Ni is the most active non-noble metal [25]. Raney nickel was introduced in the fifties as alkaline fuel cell anodes and has been used for almost 50 years as an effective electrocataliyst for cathodic hydrogen evolution [6,26]. Raney nickel is a highly porous nickel with a high internal surface area. It is obtained by alloying nickel with zinc, in which case it is also known under the name of Urushibara nickel. It can also be made out of nickel and aluminum or silicon. Zn, Al, or Si are then leached out to leave a very porous and active material. After leaching, the porous nickel coating contains hydrogen in an adsorbed state or as a nickel hydride. Therefore, it is indispensable to oxidize this hydrogen under controlled potential to obtain the activated Ra-Ni coating [27].

**Figure 4:**
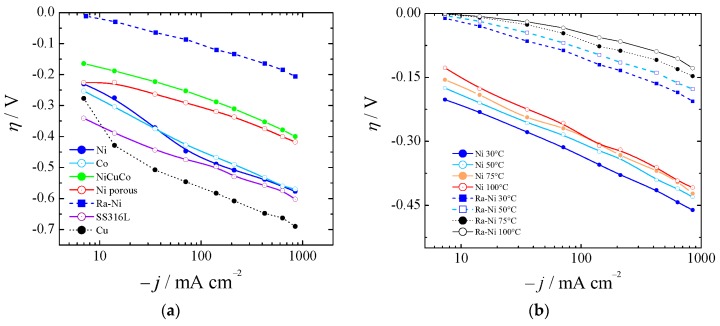
(**a**) Polarization curves for several catalysts for HER at 30 °C (Ni was not aged). (**b**) Comparison of aged Ni and Ra–Ni between 30 and 100 °C.

Polarization curves from a nickel disk electrode of 3 mm diameter were obtained, as shown in Figure 4, by working at different current densities. Raney nickel was synthesized, as described in Section 3.1, and characterized electrochemically for HER in 6 M KOH, proving its significant catalytic properties.

Figure 4a shows the HER for bare nickel (Ni), cobalt (Co), and copper (Cu), nickel-copper-cobalt alloy (NiCuCo), Raney nickel (Ra-Ni), and SS316L at 30 °C. In Figure 4b the overpotentials for aged bare Ni and Ra-Ni at different temperatures are also shown. If we compared Ra-Ni with Ni it could be seen that at 75 °C and at 400 mA cm^−2^ it was possible to reduce the cell potential by almost 300 mV.

Several other materials were taken in consideration for comparison, but not directly tested: Ni dendrites [28]; Ni-rare earth alloys [29]; NiCu compact, porous [30,31], metallurgical, and dealloyed [32,33,34,35,36,37,38]; Ni-Co-W [39]; NiCuZnB [40] smooth [22,31,41,42] and porous [43,44,45,46]; NiCo by electroless-plating deposition [47]; Ni-Co [41,43,45]; Ni-Fe [22]; in situ activation of Ni-Co with Mo [48]; electrodeposited Ni-Co-Mo; NiMo [49]; NiCoZn alloys [50,51,52]; Raney-Co [44]; Ni-Mo [53]; nonporous cauliflower-like cobalt-nickel hydroxide (CoNi) [54]; and black nickel (Ni-S) [12]. However, since their results were worse than the materials tested in this work, and the conditions of the experiments were too different from our experiments (low electrolyte concentration or uncertain equilibrium potential correction), only some of their results were summarized in Table 1, Table 2 and Table 3. As found also by other authors [21,50,55,56,57,58,59], Ra-Ni showed the best performances. Nevertheless, there is still active research on cathodic materials and their synthesis, as it is perceived that improvements are still possible in order to provide lower overpotentials and cheaper catalysts [60]. 

It can be concluded that alloying Ni with, for example, Co or/and Cu, resulted in an increased electrocatalytic activity in the HER when comparing with pure Ni, Cu, and Co. This was due to an improved intrinsic activity of the materials. However, Co or/and Cu-modification of type Raney Ni-based electrodes did not improve the apparent catalytic activity of these materials as compared to Ra-Ni. Nevertheless, it was difficult to assert whether the increase in the intrinsic activity compensated for the lower cathode surface area [57], which was one of the key features of Raney nickel.

Finally, it is important to point out that our results coincided with the ones obtained by Kjartandóttir et al. [63] for polished Ni 99% and Raney nickel obtained by physical vapor deposition or by vacuum plasma spraying [64]. It was noteworthy that Ni required an aging/activation process of some 12 h in concentrated KOH (6 M) before performing at its best. All the materials used in this study were systematically pretreated in concentrated solution before use. Without ageing, Ni behavior for HER and OER can vary up to 15% in the final overpotential. This variation can, in our opinion, explain most of the discrepancy found in in literature.

#### 4.1.2. Oxygen Evolution Reaction (OER)

The irreversibility of the oxygen electrode reaction is the main cause of efficiency losses in water electrolysis cells. It is of interest to note, as previous researchers pointed out, that, as opposed to HER, there is little difference in the polarization behavior of smooth and porous structures during O_2_ evolution. This is mainly due to ineffective utilization of the internal surface area of the porous body as the surface is blocked by bigger gas bubbles involved in O_2_ evolution [65]. 

**Figure 5:**
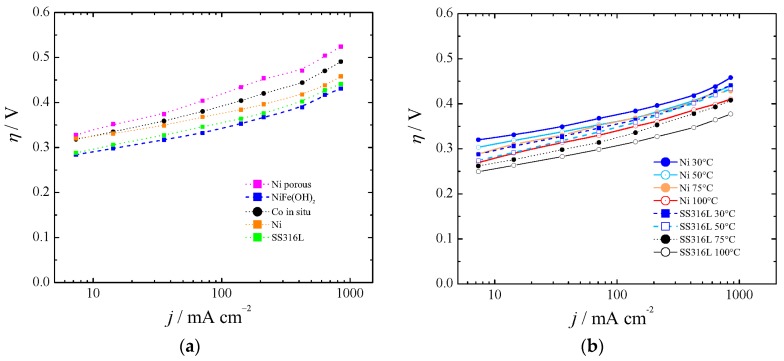
Polarization curves for the oxygen evolution reaction (OER) at (**a**) 30 °C and (**b**) between 30 and 100 °C.

As in the case of the HER cathodes, some anode materials exhibited performance variation with time. Figure 5, part (**a**) shows the polarization curves for Co in situ [47] and porous Ni, and they have been compared with bare Ni. We have synthetized Ni-Fe(OH) as described elsewhere [23,24], and found overpotentials as described by Dionigi and Strasser [66] at low temperatures (blue line in Figure 5**a**). However, we found instability at high current density, high temperature, and KOH concentration after 16 h of experiment. Hydrotalcite-wrapped Co–B alloy [67] was proposed and tested at a low current density and low OH^−^ concentration. Also, mixed oxides were investigated as electrocatalysts for OER (Fe, Co, Mo). However, due to instability or no practical gain with regards to Ni, they have been discarded. Iron impurities in thin film nickel oxides have shown good behavior in comparison with bare Ni [68], unless such low overpotentials were anomalous and could be explained by a non-proper equilibrium potential for 5.5 M KOH, as was described in Section 2.1. Hierarchically structured three-dimensional nickel–iron electrodes were suggested [69], but they did differ with regards to aged Ni if the proper equilibrium potential was used for 10 M KOH. 

Recently, it has been pointed out that commercially available stainless steel (SS) 316L can be an efficient electrocatalyst for water oxidation in alkaline solution [70]. Our results with SS316L were in agreement with in situ growth of Fe(Ni)OOH on stainless steel [71], first row transition metal (oxy)hydroxides [72], aged SS [73], or SS AISI 304 oxidized by exposure to Cl_2_ [74]. They are shown in Figure 5a for 30 °C and Figure 5b for the range 30–100 °C.

With regards to other catalysts from the literature, Co_3_O_4_ could save around 30 mV (at 400 mA cm^−2^ and 25 °C) in comparison with bare nickel metal [7]. However, it seems that it deactivates at high temperature [27], and recent studies [75,76,77,78] did not show better performance than aged SS304 [73]. Our results agreed with the ones obtained by Kjartandóttir et al. [63] for polished Ni 99% and also with the ones of Wendt [79] for smooth Ni.

Taking into account HER and OER results, it is important to point out that the present results were in agreement with those presented also by others [27,62,64] and show that OER overpotentials can be twice as large as those for HER [7,80]. As mentioned by Pletcher and Li in 2011 [6], a cell voltage target lower than 2 V at a current density of 1 A cm^−2^ for a cell operating below 100 °C is already realized. In our case, extrapolating from Figure 4b and Figure 5b, a cell comprised of Raney nickel and SS1316L would show a total kinetic overpotential of about 550 mV at 75 °C and 1 A cm^−2^.

### 4.2. Long Term Experiments and Cell Behavior

The long-term performance of a catalyst is essential for technical applications. To be able to evaluate the ageing rate of the catalyst, galvanostatic long-term experiments were carried out. In accordance with the operating conditions of commercial water electrolyzers, the electrodes were tested at a constant current density of 300 mA cm^−2^ in 6 M KOH at 75 °C.

Two run tests were performed: one with nickel in both electrodes (Ni–Ni) and the other one with SS316L on the anode and Raney nickel on the cathode (SS316L–Ra–Ni). 

**Figure 6:**
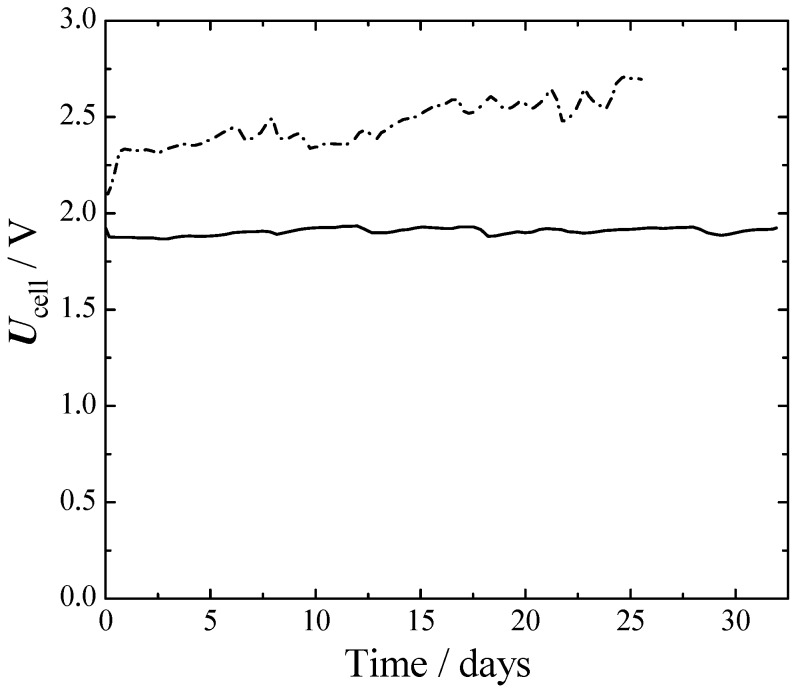
Cell voltage of long-term one-month experiments at a fixed current density and temperature. 75 °C, 1 atm. 300 mA cm^−2^. Full line—cathode: Ra-Ni, anode: SS316L. Dash dot line—cathode: aged bare-Ni, anode: aged bare-Ni.

From Figure 6 it can be seen that initially, the Ni–Ni cell had about a 250 mV larger cell voltage than SS316L–Ra–Ni. This difference grew to 650 mV at the 25th day. The better performances of SS316L–Ra–Ni were mainly due to a better HER, as can be seen from Figure 4 and Figure 5. The voltage of SS316L–Ra–Ni oscillated around 0.05 V for 30 d without giving any sign of losses during that period. Ni–Ni instead, constantly deteriorated with time.

According to literature, pure Ni for water electrolysis deteriorates mainly because of the adsorption of metallic or organic impurities that block the effective sites for HER or OER, the decrease of hydrogen adsorption capacity after it has absorbed a certain amount of hydrogen for HER [81], and the formation of surface oxide films having poor electronic conductivity for OER [13]. 

In order to evaluate the cell behavior and to compare it with bibliographic results with more optimized cell geometries, computational fluid dynamic (CFD) simulations were carried out, as described in references [8,9]. Input parameters were: the cell geometry, conductivity of the membrane-electrolyte, and kinetic behavior from Figure 4 and Figure 5. Table 4 compares the experimentally obtained average value of the cell potential (during one month), the theoretical cell potential, and the estimated *iR*, obtained by a primary current distribution (CFD) study, and finally the cell potential that would be expected with the proposed catalyst (Ra–Ni and SS316L) in a zero gap assembly.

Our results were comparable with others in similar conditions despite the different cell design [59,82,83] for SS316L–Ra–Ni and for Ni–Ni [84]. Also, these performances compared well with more advanced catalysts [79,85,86]. If we did not take into account the additional resistance of our cell design, around 0.207 Ω as estimated by the CFD result, the Raney–Ni as cathode and SS316L as anode were in agreement with the most efficient catalyst working in zero gap cells [3,79,80,87].

### 4.3. Anode Corrosion

To understand whether the catalysts showed corrosion behavior during prolonged use, an ICP-OES (inductively coupled plasma—optical emission spectrometry) analysis was carried out before and after the long-term electrolysis. Before electrolysis, there were traces of Co and Fe in solution (about 1.2 and 0.24 ppm, respectively). After long-term experiments, the amount of Cr went to 0.4 ppm and that of Fe to 0.35 ppm. Nickel was in both cases below the detection limit. It can be concluded that at a current density of 300 mA cm^−2^, concentration of 6 M KOH, and temperature of 75 °C there was no corrosion of the proposed materials at all. Our results were in agreement with corrosion studies for steel and SS316L under similar conditions [88,89].

However, in preliminary experiments with electrodes that were aged for a shorter amount of time or used directly, we noticed a slight coloration of the solution when SS316L was used as an anode. The solution turned to a very faint yellow tint during the experiment and then green at the end. Also, black powder was found accumulating on the cathode electrode. In the case of the use of SS316L, this powder was found to be magnetic suggesting magnetite content. However, black powder was found also when not working with SS316L anodes. We believe that there may be a selective or partial corrosion of the pristine electrode. However, after a while this corrosion appeared to stop. At the moment this phenomenon is still subject to research.

## 5. Conclusions

In this perspective we highlighted some of the pitfalls in the research for alkaline water electrolysis. The proper correction of the equilibrium potentials for temperature and also for electrolyte concentration is extremely important. Ohmic drop correction, which is important for high-current experiments, should be conducted carefully especially in regards to which technique was used to estimate it.

The electrocatalytic activity of Ni to be used as cathode should not be employed as a reference, as it is in general not good enough; in addition, it can show strong variations with time [81].

Whatever evaluation technique is used, polarization measurements made on fresh electrode samples after a few hours cannot be considered indicative of performance. A preactivation step, or at least long-term aging in concentrated solutions, is required.

Ni, Co, Cu, NiCu, NiCo, and NiCoCu alloys were synthetized and characterized electrochemically for HER and OER in 6 M KOH, showing important catalytic properties for commercial applications. It was demonstrated that alloying Ni with Co or/and Cu resulted in an increased electrocatalytic activity in the HER when compared to pure Ni, Cu, and Co. This was due to improved intrinsic activity of the materials. However, these improvements were not comparable with state-of-the-art Raney nickel. While, for the OER, NiFe(OH)_2_ and SS316L showed the best performances, with SS316L displaying the highest stability.

In long-term experiments, Raney-nickel with an SS316L anode outperformed a cell with symmetric nickel electrodes by more than half a volt, at 300 mA cm^−2^ and 75 °C, and showed constant voltage during the one-month experiment. Extrapolating our results to a zero-gap design, our cell had an estimated voltage of 1.7 V at 300 mA cm^−2^.

In our opinion, we are on the way to reaching the goal of accomplishing a cell voltage less than 2 V at current densities of 1 A cm^−2^ below 100 °C, as mentioned by Pletcher and Li [6].

## 6. Future Needs and Prospects

From the experience gained during our research in alkaline water electrolysis and a survey of the literature, we would like to summarize some suggestions:

(a) Show kinetic behavior with regards to overpotentials with proper corrections for the equilibrium potentials. In the present contribution we give all the tools needed to accomplish this task. In case this is not possible, remember that the total water splitting difference of potential, contrary to the single electrode potentials, shows smaller variations with temperature and electrolyte concentration.

(b) Use the appropriate technique in order to estimate the *iR* correction. We suggest AC impedance and correct it according to the electrochemical technique employed. However, when possible, minimize ohmic drop by a good cell design, with a well-positioned Luggin capillary, using concentrated electrolyte and higher temperatures.

(c) Compare results with the best-known catalyst. Bare Ni is not a good catalyst for hydrogen evolution, and it can show erratic behavior. Compare instead with a well-aged or pre-activated Raney nickel electrode.

(d) Make experiments in conditions closer to industrial situations (high electrolyte concentration and above room temperature), and test stability of the materials for at least one week.

Looking at the polarization curves, we think that the community should focus on catalysts for OER, as the overpotentials required for the same current density is at least double than that for HER. Also, to improve the efficiency of alkaline water electrolysis further, research and development efforts focusing on the development of optimized cell designs and effective electrolyte additives, use of physical/chemical electrode modifications for OER, and appropriate management of the gas bubble phenomena are still required.

## Figures and Tables

**Table 1:** Summary of NiCu alloys found in literature for HER in alkaline conditions.

	Compact and Porous NiCu [32]	Compact and Porous NiCu [33]	NiCu [34]	Dense NiCu [35]	Sintered Porous Ni-Cu [35]	Metallurgical NiCu [36]	NiCuZn [40]
*j* (mA/cm^2^)	Overpotential (mV)						
100	−410	−307	−370	−500	−400	−300	−210
200	−620	−560	−460	−650	−550	--	--
*T* (°C)	room	room	room	35	35	25	room
KOH conc.	1 M	1 M	6 M	6M	6M	6 M NaOH	1 M

**Table 2:** Summary of NiCo alloys found in literature for HER in alkaline conditions.

	Smooth NiCo [22]	Smooth NiCo [41]	Smooth NiCo [31]	NiCo [42]	Porous Ni-Co [43]	Raney Co [44]	Porous NiCo [45,46]	NiCoZn [50]	NiCoZn [51]
*j* (mA/cm^2^)			Overpotential (mV)						
100	−380	−480	−380	−430	−350	−410	−165	−125	−230
200	-	-	-	-	-	−450	−210	−145	−370
*T* (°C)	25	room	50	25	room	80	30	25	room
KOH conc.	6 M	0.5 M	30%	6 M	30%	3 M	30%	0.5M NaOH	1 M

**Table 3:** Best alloys for HER found in the literature.

	Ni- Porous [31]	Ni_2_PW_12_ [44]	Ni-Porous [61]	Ra–Ni [30] from Al	Ra–Ni [55,62] from Al and Ti	Ra–Ni [57] from Zn
*j* (mA/cm^2^)	Overpotential (mV)					
100	−300	−300	−150	−115	−110	−110
200	--	−365	−190	−170	−160	−150
*T* (°C)	50	80	30	22	30	50
KOH conc.	30%	3 M	30%	6 M	1 M	30%

**Table 4:** Theoretical and experimental cell voltage using Ra–Ni and SS316L electrodes (75 °C, 1 atm. 300 mA cm^−2^).

*U*_cell_ Exp/V	*U*_cell_ Theo/V	*R*_cell_/Ω	*U*_cell_ Zero-Gap/V
1.905	1.935	0.207	1.705
